# The Swedish Perioperative Register: Description, validation of data mapping and utility

**DOI:** 10.1111/aas.14174

**Published:** 2022-12-14

**Authors:** Björn Holmström, Gunnar Enlund, Peter Spetz, Claes Frostell

**Affiliations:** ^1^ Department of Medical Technology, Development and Management Karolinska University Hospital Stockholm Sweden; ^2^ Department of Anaesthesia and Intensive Care Uppsala University Hospital Uppsala Sweden; ^3^ Department of Anaesthesia and Intensive Care Mälarsjukhuset Eskilstuna Sweden; ^4^ Department of Anaesthesia and Intensive Care Karolinska Institute at Danderyd Hospital Stockholm Sweden

**Keywords:** anaesthesia, complication, COVID‐19, perioperative medicine, quality register, surgery

## Abstract

**Background:**

Since 2013 surgical units in Sweden have reported procedures to the national Swedish Perioperative Register (SPOR). More than four million cases have been documented. Data consist of patient ID, type of surgery, diagnoses, time stamps during the perioperative process (from the decision to operate to the time of discharge from the postoperative recovery area) and quality measures. This article aims to describe SPOR and validate data mapping. Also, we wished to illustrate the utility of the SPOR in assessing variations in national surgical capacity during the COVID‐19 pandemia years 2020–2021.

**Methods:**

After a detailed description of SPOR, we report on the validation of data performed by comparing data from local databases with data stored in the central SPOR database, assessing missing values and accuracy. Effects of the pandemic on surgical capacity were described by developing an index, based on the number of performed surgical procedures per week during four production weeks in January 2020. Subsequent weeks were then compared with this baseline.

**Results:**

The validation effort demonstrated nearly 100% data accuracy for the number and type of surgical procedures between local and central data. Missing data was a problem for some parameters. The number of performed surgical procedures decreased dramatically from week 11 in 2020 compared with normal production on a national basis, mainly impairing elective surgery.

**Discussion:**

Data validation revealed good agreement between local and central databases. The changes in national surgical capacity during the pandemic were illustrated by an index based on the reported surgical production.

**Editorial Comment**

This special article introduces a national registry for perioperative cases, factors and events for Sweden, where most health care is managed in regional public health care systems, and these are almost all succeeding in providing their data to this database.

## 1 INTRODUCTION

There has been an unmet need to provide inexpensive and easily accessible data on anaesthesia and surgical health care in Sweden. Many stakeholders can be identified, including the surgical and anaesthesia professions, academia, healthcare providers, patients and the broader community. In addition, modern health care generates and consumes data to monitor quality and facilitate improvements in care. At present, there are over 100 active quality registers in Sweden. The Swedish PeriOperative Register (SPOR, Swe: *Svenskt PeriOperativt Register*) was launched in 2011. The main goal of this project was to gather data from each performed surgical intervention through the automatic transfer of information already sampled in the local operation‐planning system, creating a national database at the Uppsala Clinical Research Centre (UCR) in Uppsala, Sweden.

Patient‐related surgical quality can, in general, be measured in different dimensions from a patient's perspective.

First, for patients who have undergone surgery, quality can consist of whether the surgery was performed successfully, without perioperative or postoperative complications, without causing severe postoperative pain or postoperative nausea and vomiting (PONV), and with as brief a postoperative rehabilitation period as possible. Second, for patients who have not yet undergone surgery, quality can be measured by the time between the decision to operate and the start of surgery. Per this measurement, shorter waiting periods indicate better quality. Before the SPOR, there was no way to quantify either of these aspects of patient‐related surgical quality, at least not on a national basis.

Quality from a registry perspective means the data extracted from a national database are as accurate as possible. Additionally, quality per healthcare leadership could involve effective throughput of each patient category, at a reasonable cost and with few avoidable complications. Each stakeholder may have a unique perspective here.

### 1.1 Aims

The first aim of this article is to describe the development and maintenance of SPOR in detail. In an editorial, SPOR was just described in brief.^1^ In previously published articles based on SPOR data, this information has not been presented.^2^, ^3^, ^4^

A second aim is to verify the accuracy of this database information, supporting its use in the assessment of national surgical capacity or for daily planning in surgical theatres. We have chosen to compare source data in local databases at participating surgical units with the data stored in the national SPOR database.

Furthermore, to illustrate the utility of aggregated SPOR data, we have used a SPOR‐index to follow the effects on national surgical production during the COVID‐19 pandemic.

## 2 BACKGROUND

Before the establishment of SPOR, there was no practical way to answer questions like ‘How many surgical theatres are open in Sweden this week?’, ‘What are the 30‐ and 90‐day mortality rates of elective gall bladder surgery in Sweden for ASA II patients?’ or ‘Is there a regional variation in outcomes between acute and elective caesarean sections?’

The need to provide such quantifications drove attempts in the 1990s and early 2000s to establish a truly national, comprehensive and effective register. However, these first attempts proved unfeasible, as they were based on telephone inquiries and manual registration that required overwhelming labour. In 2010 it was generally agreed that a digital approach would be the most economic and practical option.

The SPOR was launched in 2011 with the financial and organizational support of the Swedish Society for Anaesthesiology and Intensive Care Medicine (SFAI).

A board was selected to establish the minimum requirements for this endeavour. Per their directive, the resulting SPOR should have:

- mainly automatic data capturing from local, computer‐based operational planning systems (OPSs)
- regular access to Swedish population indexes such as the Cause of Death Register, giving automatic updates on mortality
- pseudo‐anonymizing of individual data by assigning a unique treatment number to each surgical procedure registered in the database
- assumed consent from every patient to collect pseudo‐anonymized data but offering an opt‐out possibility at any point, for any reason
- certification of taxonomy for each parameter registered and collected via SNOMED CT (Systematized Nomenclature of Medicine Clinical Terms) for content and definition^5^
- automatic validation and a local feedback loop of corrupt data for correcting in or discarding from the local database

After thorough infrastructural planning and the design of local and central databases, including mapping of all variables, the first registrations were collected in December 2013.

### 2.1 A brief technical description of SPOR (for more details see the Technical Supplement)

The *perioperative process* has been defined in SPOR as the period from the decision to operate until the operation has been performed and the patient is discharged from postoperative care. The process occurs in four phases: operation notification, planning, surgery and postoperative care. Each phase includes several well‐defined variables, some of which are mandatory. The variables are grouped according to these four phases. Certain basic data about each patient (e.g., date of birth, Swedish personal identity number or coordination number, administrative gender) are mandatory.

The variables consist of patient data, time stamps, codes for the surgical procedure and diagnoses. Outcomes and quality parameters (e.g., perioperative bleeding, patient body temperature, postoperative pain, nausea/vomiting and selected vital parameters) are also reported. Every night, surgical units report their activity—consisting of all surgical interventions performed during the last 24 h with these variables—to the SPOR. The variable list contains parameters that can be used to assess quality and production. With daily reporting from all publicly run hospitals, SPOR offers a close overview of Sweden's surgical production.

Using a variable list entitled SPOR version 1, the central database started gathering information in 2013, containing 71 variables at the time. Due to continuous updates of the variable list through 2020, the current SPOR version 4 (SPOR 4.0) now contains a total of 159 variables.^6^ All variables have (or have been requested to receive) a SNOMED CT classification. Follow‐up data include the Swedish‐validated versions of Quality of Recovery 15 (QoR15) and Post‐Anaesthesia Workload Instrument (PAWI) for people over the age of 18.^7^, ^8^

As soon as a decision to operate is registered in an OPS, the data transfer can begin; this information is supplemented when updates occur in the planning, operation or postoperative phase. With SPOR 4.0, there is an opportunity to review not only the operations/procedures performed but also the waiting list that shows anticipated needs.

This system supports the automatic transfer of data from a journal system to SPOR's database.

Technically, this task is solved with an Extensible Markup Language (XML) scheme^9^ that describes in detail what each variable should look like to be transferred correctly.

For missing definitions or standards, the SPOR sets qualifications, as decided at user meetings twice a year. If all the required fields are not met, the record is not accepted and is returned. The received records are checked to ensure they meet the structure and limitations of the XML scheme and against 52 logistic controls, including 130 variable combinations. Any error encountered moves to a correction error list and the record is not used in reports until all errors are corrected. Corrections take place in an OPS (i.e., the basic journal), and the record is then re‐sent. In this way, the basic data at the sending unit will always be corrected. This routine has validated and corrected all the local databases at participating hospitals.

The database is also designed to support the regular, rapid transfer and updating of data between systems. Currently, SPOR receives about 50,000 new or updated entries each night.

To transfer and receive data and extract reports from SPOR, a high‐security login credential is required. SPOR uses a SITHS‐card (Safe IT Health and Medical Care) login.

As a central national database, SPOR has successively grown from 2013 until now. In early 2022, SPOR had assembled data on over 4 million surgical procedures from all government‐funded Swedish hospitals except two. Some private hospitals also report their production volumes to the SPOR. So far, about 30 requests for data export from the SPOR national database to research groups and a few government agencies have been served. Our log at UCR over locally performed data exports rose above 100,000 different extracts in 2022.

## 3 METHODS

### 3.1 Validation of data quality

The SPOR board sought to ascertain that process data such as dates, reported times and the main surgical code were correctly reported and stored in the central database. In other words, the mapping procedure itself was targeted for validation. An ethics permission process was completed, and approval was secured in 2018 (Ethics Review Board, Uppsala Nr 2018‐043).

By 2015, the UCR had been asked to choose *n* = 40 treatment numbers randomly from six reporting surgical units that had volunteered to participate in a data‐validation process. We chose 23 obligatory parameters to be collected for each of these 240 procedures.

In the second step, these 240 treatment numbers were sent to the locally responsible individual at the hospital in question (40 to each surgical unit), with a request to enter the locally recorded value on the parameter, including a ‘missing value’ label if applicable, for each parameter. The parameter sheet was returned on a USB drive or digitally as a crypto file. Finally, one of the authors (CF) manually compared the reported values for each parameter with the value in the central SPOR database at UCR. After identifying missing data or discrepancies, each participating surgical unit was asked one additional time about the local data on these data positions.

Of these procedures, 120 were assigned to be checked via re‐validation in 2019. This additional step aimed to assess whether data collected and stored in the central SPOR database became corrupted or deleted over time.

### 3.2 SPOR index

Over the last few years, a growing number of Swedish hospitals have begun to report all their surgical procedures to the SPOR database. In the early spring of 2020, all Swedish hospitals except two were reporting. This enabled us to determine that the reported number of surgical procedures during 4 weeks from January to February 2020 could be seen as the total pre‐pandemic capacity for surgery in Sweden across publicly financed and produced health care. Based on this assumption, G. Enlund, a member of the SPOR board, launched the concept of a ‘SPOR index’ set at 100% for these 4 weeks.

The utility of this index became clear when the performed surgical procedures from the reporting units rapidly decreased at the beginning of March 2020, due to the COVID‐19 pandemic. From that point onward, the SPOR‐index values compared with ‘100%’ have continuously been reported in the public domain every week.

## 4 RESULTS

### 4.1 Validation

Table 1 states in real numbers and percentages the degree of agreement between the local database and the central SPOR database, for a total of 13 parameters with relevance for the validity of the SPOR index and logistics.

**TABLE 1:** Results of 13 requested variables, comparing values in the local operating planning system at six hospitals with the national SPOR database

Variable	Requested	Delivered Response	Missing Value	Agreement, *n*	Agreement %
Date of procedure	210	210	0	210	100
Main surgical code	210	210	0	197^a^	94^a^
Additional surgical code	210	55	155	52	96
Anaesthesia code	210	207	3	205	99
Anaesthesia code—additional	210	79	131	72	91
Perioperative complication	210	153	57	122	80
Postoperative complication^a^	210	133	77	118	89
Time stamp—start of process^b^	210	210	0	209	99.6
Time stamp—start of anaesthesia	210	210	0	209	99.6
Time stamp—start of surgery	210	210	0	210	100
Time stamp—end of surgery	210	210	0	209	99.6
Time stamp—out of OR	210	210	0	206	98
Time stamp—out of postop^c^	210	177	33	166	94

*Note*: We requested data from each contributing surgical unit, and each delivered response reflects the data sent back after local manual extraction; a ‘missing value’ is a data point with no value stated, and ‘agreement’ (*n* and %) shows the comparison between the value in the central database compared with a delivered response. Thus, it is possible to have 100% agreement on delivered data, even with missing values.

^a^ See Section 4.

^b^ ‘Time stamp – start of process’ = when the patient arrives in the surgical area, with anaesthesia staff taking responsibility.

^c^ Several patients had no recording of any postop stay (minor procedures).

We received a complete dataset from five of the six surgical units. The last unit chosen misunderstood the instructions and ran a different validation process of their own. In the end, we only obtained data on 10 treatment numbers from that unit, which resulted in a complete returned dataset of *n* = 210 treatment numbers from six surgical units. Most identified discrepancies (about 2% of the total) represented errors in manually copying local unit data into the provided Excel datasheet.

As seen in Table 1, there was good to excellent agreement on each logistics parameter, such as the date of the procedure, the main surgical code before the procedure and the six timestamps selected for analysis. There was also good agreement (94%) between the planned and later reported surgical code, except for 13 treatment numbers. However, this discrepancy was almost always explained as a correct reporting of a disease process, where a planned surgical intervention was slightly altered due to developments after a decision to operate had been made. As two examples, a planned *ligation of v. saphena magna* was postoperatively reported as *ligation of a vein*, and a planned *proctoscopy* was postoperatively reported as *endoscopic rectal treatment*. Both these alterations resulted in a different postoperative main surgical code. In contrast, some parameters displayed lower agreement and accuracy and/or more missing values. Of note, there were a few cases in which the postoperative period was described as spent in an ICU, although no perioperative complication had been observed.

In a few cases, a secondary surgical code was added after surgery in the local OPS but not necessarily noted in the patient charts. With reported types of anaesthesia, a confusing picture sometimes occurred. For example, in one case, only the code for oxygen saturation measurements with a pulse oximeter was registered in addition to local anaesthesia, with the pulse oximeter code appearing as the ‘main anaesthesia procedure.’ In addition, reported ASA risk classification data at the decision of surgery displayed 20% missing values.

Furthermore, all six surgical units examined in this study demonstrated incomplete datasets for their 2015 postoperative period: in 16% of procedures, no timestamp data were recorded after the end of surgery and in 37% no information about postoperative complications (yes/no) was entered. This included a lack of data on pain scoring, PONV scoring and more. Data from 2019 were sometimes more reassuring and complete but still showed a considerable lack of consistent documentation.

The re‐validation step performed about 3 years later for the same treatment numbers demonstrated >98% agreement between the first and later times in the central SPOR database. Most identified deviations were due to an earlier ‘missing value’ being replaced by data. This can be explained by the subsequent regular overwrite function, enabling the local database to be adequately mirrored in the central SPOR database at UCR to incorporate data with a lag time.

### 4.2 SPOR index

**FIGURE 1:**
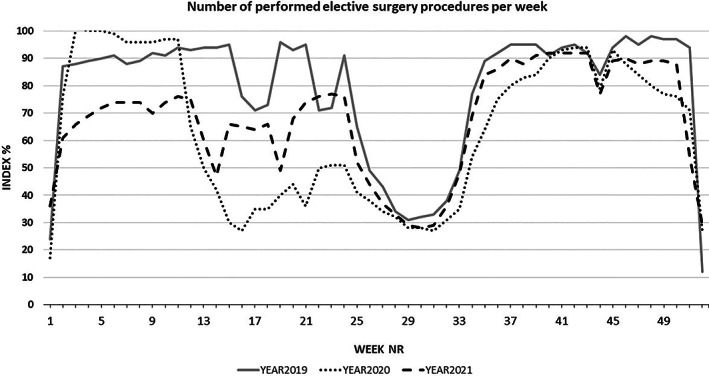
Production of elective surgical procedures performed in Sweden from 2019 to 2021, demonstrated as a percentage of the production of weeks 3–6 in 2020 (=SPOR index 100%). The diagram shows the ‘natural’ reduction in procedures during the Christmas/New Year holidays, summer holidays and week 44, which is a school holiday in Sweden. Additionally, the diagram contains some low‐production weeks (e.g., holidays beginning on a Thursday, which result in a 3‐day production week). The diagram demonstrates the rapid reduction in number of performed procedures from week 11 of 2020 until summer 2020 (COVID‐19 phase 1), during autumn 2020 (COVID‐19 phase 2) and during spring 2021 (COVID‐19 phase 3).

**FIGURE 2:**
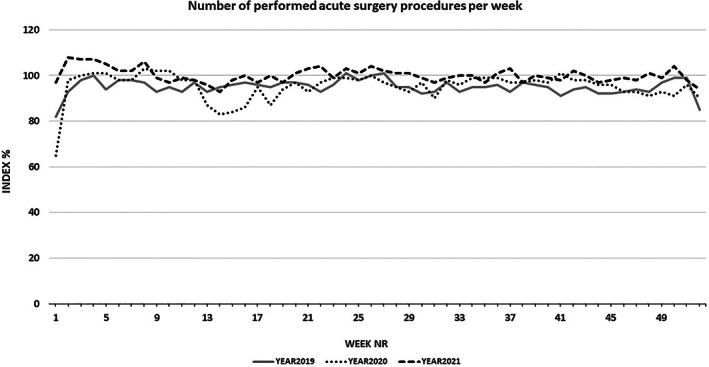
Production of acute surgical procedures performed in Sweden from 2019 to 2021, demonstrated as a percentage of the production of weeks 3–6 in 2020 (=SPOR index 100%). There is no large effect on the number of performed procedures, apart from a few weeks after week 11 of 2020. Even during vacation periods, Swedish hospitals keep a stable level of capacity for acute surgery.

Compared with the assumed maximal capacity (SPOR index = 100%), the SPOR index for reporting Swedish hospitals has continuously been lower than 100% for elective surgery for more than 80 weeks since March 2020 (Figure 1). However, as demonstrated in Figure 2, the number of performed surgical cases labelled ‘acute’ was not affected to the same magnitude.

## 5 DISCUSSION

This article describes the SPOR initiative, outlines a validation effort and demonstrates how SPOR data can be employed as a real‐time tool for examining daily planning and performance in surgical theatres. It has also allowed us to follow the impact of a pandemic on Swedish national and regional surgical capacity over time. In short, by utilizing SPOR, we identified a massive impact of the COVID‐19 pandemic on the production of Swedish elective surgery from 2020 to 2021. Next, we will discuss our SPOR initiative, present and future utility and findings during the pandemic, point by point.

### 5.1 Context

The SPOR allows member surgical production sites to report data continuously from their various electronic surgical planning systems. The data are first automatically validated for inconsistencies and erroneous data entries and then aggregated in the national SPOR database at UCR. With those tasks accomplished, member clinics can scan, analyse or download their local data at will. They may also conduct benchmarking with pseudo‐anonymized data in comparison to elected clinical surgery departments in Sweden. We consider these features to be a uniquely quality‐enhancing capacity for Swedish surgical production sites attached to the SPOR.

### 5.2 Validation

Although there are published articles based on data extracted from the SPOR database,^4^, ^10^, ^11^, ^12^ this is the first study systematically examining the validity of at least some of the data collected locally and then automatically mapped and transferred to a national database. Reassuringly, several key variables did reach a high degree of agreement between a local source database and the national database at UCR. Among them was the correct main surgical procedure of utmost importance, as well as all logistics parameters such as the date of the procedure and recorded main time points (i.e., start and finish of surgery, start and finish of anaesthesia, start and end of perioperative care in the surgical area). Other registers have also examined the completeness and accuracy of their assembled data.^13^ We note that an agreement per parameter of >95% can be considered a high level of accuracy.

Provided that researchers can maintain and demonstrate similarly good performance of other variables, the SPOR has the potential to be a useful tool for easy and low‐cost data collection from anaesthesia and surgical care. This would facilitate clinical work aimed at testing and implementing modified or new improved procedures, during the surgical and postoperative care process. We have already seen multiple examples shared among surgical units using SPOR data to monitor the effects of changes in the perioperative process.

### 5.3 ICU postoperative care

Immature and local applications of SPOR have created challenges with aggregating data meaningfully to a higher national level. For example, several smaller hospitals regularly use space adjacent to the ICU for uncomplicated postoperative care, especially during nights and weekends. Such areas are usually run by ICU‐employed staff. This resulted in misleading reports that patients without complications had required ICU care postoperatively, which took us some time to understand and define correctly.

### 5.4 ASA score as a missing value

We could note that scoring for ASA was absent for several procedures (about 20%) studied from 2015 to 2016. Erroneous mapping or immature clinical routines (e.g., the ASA score perhaps noted on article but not electronically entered into the OPS) were among the reasons found.

### 5.5 Perioperative and postoperative complications

Data recorded from several surgical procedures did not state whether any complication was noted during the perioperative process. For surgical procedures studied in 2018 and 2019, such information was still often lacking. Thus, the SPOR cannot yet be used with high accuracy for studies of the observed‐versus‐predicted risk of mortality and morbidity, especially for a subset of surgical procedures lacking ASA classification and/or notes on complications.

It will remain a major future challenge to inform all staff and healthcare leaders of the vital need to improve the register's data quality by entering all required information at each local surgical unit. This effort is necessary for the true potential value of SPOR to be realized. There will always be the possibility that locally enforced interpretations and routines could dominate and corrupt an aggregated higher level of analysis. Only the central SPOR leadership can offer direction by identifying and enforcing more rigorous data reporting.

Nevertheless, the positive findings in this validation support the usefulness of SPOR as a tool to describe the burden of disease and care in relation to all publicly funded and performed diagnostic categories of surgery in Sweden.

### 5.6 SPOR during the COVID‐19 pandemic

As the number of infected patients admitted to inpatient care in Sweden increased almost exponentially in early March 2020, many Swedish hospitals implemented a strategy to create a maximum capacity for intensive care of COVID‐19 patients. This limited the capacity for surgery, as areas usually reserved for postoperative care were converted to temporary COVID‐19 ICUs. At several hospitals, even surgical theatres were converted to intensive care facilities and equipped with patient beds, ICU ventilators and monitors. This adaptation also allowed for the transfer of specialized healthcare personnel such as anaesthetic nurses and clinicians from the surgical theatres to ICU wards. Even several surgeons and surgical assistants transitioned to ICU care during the peak impact of COVID‐19 in Sweden in late spring 2020. This echoes reports from other countries' experiences with the first waves of the pandemic.^14^, ^15^, ^16^, ^17^

Using SPOR data, we could show that during the pandemic, surgical procedures labelled as ‘acute’ were still performed in similar numbers as during the pre‐pandemic period (Figure 2). Thus, the reduced surgical resources were prioritized for these patients. This chosen strategy resembles the prioritization observed and reported in other studies.^18^, ^19^, ^20^, ^21^, ^22^

Furthermore, based on an analysis of preliminary data from the online reports retrievable from the SPOR database, spot‐checks for some diagnoses indicate the patient groups with postponed surgery during this period were primarily those with benign or non‐life‐threatening symptoms, such as pain from joint arthrosis or ENT problems in smaller children (Peter Spetz, personal communication).^22^ Studies are needed to validate these theories and analyse the prioritization of surgical cancer cases during the COVID‐19 pandemic.^23^

One limitation of the SPOR initiative is that it does not currently cover production at smaller, privately owned surgical units. This is mainly because they cannot report to SPOR, as they lack operation planning systems that are compatible with the SPOR interface. Work is in progress to overcome this limitation, but at this point, we cannot state anything about their activity in relation to the pandemic using SPOR data.

## CONFLICT OF INTEREST

All authors are members of the SPOR board.

## Supporting information


**Appendix S1.** Supporting Information.
