# Supplementary material for: The Swedish Perioperative Register: Description, validation of data mapping and utility
Source: Acta Anaesthesiol Scand. 2022 Dec 14;67(2):233–9. doi: 10.1111/aas.14174 (PMC10108284; doi:10.1111/aas.14174)
Supplement: Supplementary file 1 — Appendix S1. Supporting Information. [file AAS-67-233-s001.docx]

# Technical supplement to:

# **The Swedish Perioperative Register (SPOR):** *description, validation of data mapping and utility*

### What is the SPOR?

The SPOR is one of over one hundred national quality registers within the Swedish healthcare system. It is among the few registers capturing data from a cohesive healthcare process, rather than from patients with a certain diagnosis or specific medical treatments.

### How is the SPOR run?

SPOR has a steering committee of around 10 persons, elected at each annual meeting of members. Funding consists of government means and membership fees, along with a separate fee for each reported treatment. The steering committee works closely with the staff of Uppsala Clinical Research Centre (UCR), who run the actual SPOR database. In monthly meetings, the members of the steering committee decide which steps are necessary for SPOR development, such as additional variables or new definitions, as well as improved or new reports.

### What does the SPOR contain?

In the SPOR, the perioperative process starts with a decision to operate in the medical record systems, continues while the surgery is performed, and ends when the patient is discharged from postoperative care. The process has been divided into four different phases:

- decision to operate
- planning
- surgery
- postoperative care

Within each phase, there are several well-defined variables, some of which are mandatory in each report to the SPOR.(1)

Administratively, there is an initial information structure with legal and organizational basic data regarding the reporting entity, including organizational affiliation such as region, administration, hospital, surgical unit, and clinic. In the upcoming version 4.1 (2022), reports also note whether each reporting unit is publicly and/or privately funded.

Certain basic data about the patient are mandatory (e.g., date of birth, Swedish personal identity number or coordination number, and administrative gender). Each reported treatment gets a unique treatment number, which identifies the treatment report in the database.

The variable list contains parameters that can be used for quality and production assessments. With the daily reporting from all publicly run hospitals, SPOR offers an overview of Sweden's surgical production in essentially real time.

With the variable list ´SPOR version 1.0´, the central database started capturing data in 2013, containing 71 variables. Following continuous updates through 2020 with SPOR version 4 (SPOR 4.0), it now contains a total of 159 variables. The SPOR version 4.0 contains 110 variables for perioperative recordings, 28 variables for postoperative follow-up, and 21 calculated or government-imported variables. All variables have (or have been requested to receive) a SNOMED CT classification.(2)

SPOR is a member of Register Utiliser Tools (RUT), which is managed by Vetenskapsrådet (Swedish Research Council).

Follow-up data include the Swedish-validated version of Quality of Recovery 15 (QoR-15) (3) and Post-Anaesthesia Workload Instrument (PAWI) for patients over the age of 18.(4)

From different Swedish government registers, there is also an automatic connection to address registers with regional and municipal affiliation and registration of date of death where appropriate.

As soon as a decision to operate is registered in a digital operation planning system (OPS), the transfer can begin, and it is then continuously supplemented when updates occur in the planning, operations, or postoperative phase.

Therefore, SPOR 4.0 provides an overview not only of the operations/procedures performed but also of future needs (i.e., waiting list).

### Technical description of links to OPS, mapping, and transfer to SPOR

The register offers built-in quality controls, error lists, and handling of corrupt records.

At its most basic level, this system involves the automatic transfer of digital data from a journal system to SPOR's database. A separate manual input and manual transfer process would cost the healthcare system many hours of work, as more than 650,000 operations take place annually in Swedish hospitals. A general assessment is that a corresponding manual input/transfer would cost more than 120 full-time positions annually (about 20 minutes per operation). Technically, this is solved with an Extensible Markup Language (XML) schema that describes in detail what each variable should look like to be transferred correctly.

Some restrictions exist concerning the text, format, and numerical size or fulfilment of codework standards such as ICD (International Classification of Diseases), KVÅ (Classification of care measures), KMÅ (Classification of medical measures), and SKV704 (Tax Agency brochure 704 for a description of social security number). If there are missing definitions or standards, SPOR has developed its own, which have generally been accepted to apply nationwide.

The data are sent with strong authentication to UCR, one of six official nodes in Sweden for receiving and handling high-quality health data.

When a technical connection (‘mapping’) has been made of the variables that will be sent from the OPS to SPOR, an initial transfer to a UCR test database takes place to validate the mandatory requirements and the quality of the submitted data. Several of the most common OPS have developed export modules.

The received records are checked to ensure they all meet the structure and limitations of the XML schema and against 52 logistic controls, including 130 variable combinations. If all requirements are not met, the record is returned and not accepted. Any errors are entered in a correction list, and the record is not used in reports until all errors have been corrected. Correction takes place in the local OPS, and the record is then sent again. In this way, the basic data at the sending unit will always be corrected.

The test database also checks organizational affiliations, naming units for internal use via HSA-ID (Health Care Address Register – Identification) for units as well as for persons, and naming in plain language for upcoming reports. In addition, the connection between certain fields is verified to meet the requirements specified by the SPOR. After repeated checks in the test database show a well-functioning data capture, transfer to the production database can begin.

In the production database, all the checks mentioned above are performed at all update times. An additional 9 checks are made on arrival to the production database. From 2014 to 2019, another 45 deep dives into the production database were made to improve quality.

A record can be updated an unlimited number of times by sending all data (new and old) again and then overwriting the already-registered data. Registration of the date of the first and last upload is available.

A corrupt record that cannot be corrected is transferred to a special location in the database to be used in calculating the loss of data withdrawal.

At a similarly basic level, the system supports the regular, fast transfer and updating of data between the systems. Today, SPOR receives about 50,000 new or updated entries each night. Usually, the transfer takes place before 4:30 a.m. so that, after checks and background runs, data are presentable in reports from 6:30 a.m. onward and contain all the correct data from the previous day and backward. Initially, some OPS transferred data batch-wise every week to month, but now all data automatically are transmitted each night.

### Twin reporting systems: login-based and open-access

To transfer and receive data and take reports from SPOR, a secure login is required. SPOR uses a SITHS (Safe IT Health and Medical Care) card login and is approved by Swedish healthcare governance for national healthcare systems. The SITHS card is used by Swedish healthcare professionals and acts as an e-ID. e-ID uses two-factor authentication and therefore meets the requirements for strong authentication. Each legally delimited entity decides which employees will have access to the reporting system.

After signing in, a regular user of reports can see all participating units’ fixed reports. For legal reasons, a few special reports may only be downloaded by certain named persons from each legally delimited entity. This applies, among other things, to the report in which all submitted data—structured, processed, and supplemented—can be returned to the incoming unit for local processing.

The SPOR's aim is that all permanent regular reports—currently more than 40—can eventually be outsourced for open publication, but currently Swedish laws do not allow this. A few SPOR reports are already available in Open Comparisons of Health Care, published by the National Board of Health and Welfare and available on [www.SPOR.se](http://www.SPOR.se).

All permanent reports can be locally configurated in several variations, such as the period of search, inpatient or outpatient, acute or elective, type of operation (KVÅ/KMÅ code or part of code-string), and much more. For comparison with other hospitals, most of the permanent reports can be shown with the results for the whole of Sweden or a similar group of hospitals as the current selection. Until March 2022, more than 100.000 reports have been downloaded for local follow-up and quality improvement.

Furthermore, the SPOR has developed three indexes for open comparison between Swedish hospitals. The SPOR Quality Index contains the results for nine variables for hospitals with both acute and elective surgery or six variables for hospitals with only elective surgery. The Degree of Completeness Index describes the degree to which every unit has filled the values of chosen, important variables. Finally, the SPOR index demonstrates the effect of the Covid-19 pandemic on Swedish surgical capacity.

## References

1. <https://spor.se/wp-content/uploads/2020/09/SPOR-4.0_Variabellista_Original_20-09-30.xlsx> [

2. <https://www.assess-ct.eu/home/> [

3. Lyckner S, Böregård IL, Zetterlund EL, Chew MS. Validation of the Swedish version of Quality of Recovery score -15: a multicentre, cohort study. Acta Anaesthesiol Scand. 2018;62(7):893-902.

4. Idoffsson Å, Olsson C, Holmén A, Granberg-Axell A, Chew MS. Development and validation of an instrument to measure nursing workload in the postanaesthesia care unit: An observational study. Eur J Anaesthesiol. 2020;37(10):864-73.
